# Supplementary material for: MITOL prevents ER stress‐induced apoptosis by IRE1α ubiquitylation at ER–mitochondria contact sites
Source: EMBO J. 2019 Jun 13;38(15):e100999. doi: 10.15252/embj.2018100999 (PMC6669929; doi:10.15252/embj.2018100999)
Supplement: Supplementary file 1 — Expanded View Figures PDF [file EMBJ-38-e100999-s001.pdf]

Expanded View Figures

Figure EV1. Acute loss of MITOL leads to abnormal ER morphology independent of mitochondrial damage, related to Fig 1.

A, B Morphological classification of mitochondria or the ER. The morphology of either mitochondria (A) or the ER (B) is classified as normal or abnormal. Mitochondrial network is visualized by anti-Tom20 staining. To clarify the ER network, MEFs were transfected with mCherry-Sec61β (mC-Sec61β) 24 h prior to analysis. The lower panels show fivefold (A) or 4.5-fold (B) magnification images of the boxed regions. Arrowheads indicate abnormal parts of the ER network.

C Mitochondrial fragmentation induced by acute MITOL knockout. $MITOL^{+/+}$ or $MITOL^{F/F}$ MEFs were treated with ethanol (EtOH) as control or 0.4 μM 4-hydroxytamoxifen (4-OHT) for 2 days, and then, MEFs were incubated in fresh medium for 2 days (A–E). All experiments using 4-OHT below were performed at concentration of 0.4 μM. MEFs were immunostained with anti-Tom20 antibody. The lower panels show fivefold magnification images of the boxed regions. Percentages of cells with abnormal mitochondria were calculated from 100 cells by visual inspection in each independent experiment. Scale bar represents 10 μm. Error bars represent SD (n = 3). **P < 0.01 (Student's t-test). $MITOL^{+/+}$: $MITOL^{+/+,\ ERT2\text{-}Cre}$, $MITOL^{F/F}$: $MITOL^{Flox/Flox,\ ERT2\text{-}Cre}$.

D Abnormal ER morphology induced by acute MITOL knockout. MEFs treated with EtOH or 4-OHT were transfected with mC-Sec61β 24 h before analysis. The lower panels show fivefold magnification images of the boxed regions. Arrowheads indicate abnormal parts of the ER network. Percentages of cells with abnormal ER were calculated from 50 cells by visual inspection in each independent experiment. Scale bar represents 10 μm. Error bars represent SD (n = 3). *P < 0.05 (Student's t-test).

E, F Effects of two shDrp1 on Drp1 expression and mitochondrial morphology in acute MITOL-KO MEFs. MEFs treated with indicated reagents were transfected with each shDrp1 vector or shGFP 24 h before immunoblot analysis with indicated antibodies (E) or immunofluorescent analysis with anti-Tom20 antibody (F). The lower panels show fivefold magnification image of the boxed regions. Percentages of cells with abnormal mitochondria were calculated from 50 cells by visual inspection in each independent experiment. Scale bar represents 10 μm. Error bars represent SD (n = 3). *P < 0.05 (Student's t-test).

G Drp1 repression did not resolve abnormal ER morphology in acute MITOL-KO MEFs. MEFs treated with indicated reagents were transfected with both mC-Sec61β and indicated shRNA vectors for 24 h before the observation of ER morphology. The lower panels show fivefold magnification image of the boxed regions. Arrowheads indicate abnormal ER. Percentages of cells with abnormal ER were calculated from 50 cells by visual inspection in each independent experiment. Scale bar represents 10 μm. Error bars represent SD (n = 3). n.s.: not significant (Student's t-test).

H, I Chronic knockout of MITOL exhibited abnormal ER morphology. Chronic MITOL-KO MEFs were immunostained with anti-Tom20 antibody (H). MEFs were transfected with mC-Sec61β 24 h before analysis (I). The lower panels show fivefold magnification image of the boxed regions. Arrowheads indicate abnormal parts of the ER network. Percentages of cells with abnormal mitochondria or ER were calculated from 100 (H) or 50 cells (I) by visual inspection in each independent experiment. Scale bar represents 10 μm. Error bars represent SD (n = 3). *P < 0.05, n.s.: not significant (Student's t-test).

J Increased resting Ca^{2+} in the ER of MITOL-KO MEFs. MEFs were transfected with G-CEPIA1er, Ca^{2+} indicator for the ER, 24 h before analysis. Resting Ca^{2+} in the ER was calculated from 10 cells in each independent experiment as described in methods. Error bars represent SD (n = 3). *P < 0.05 (Student's t-test).

© 2019 The Authors

© 2019 The Authors

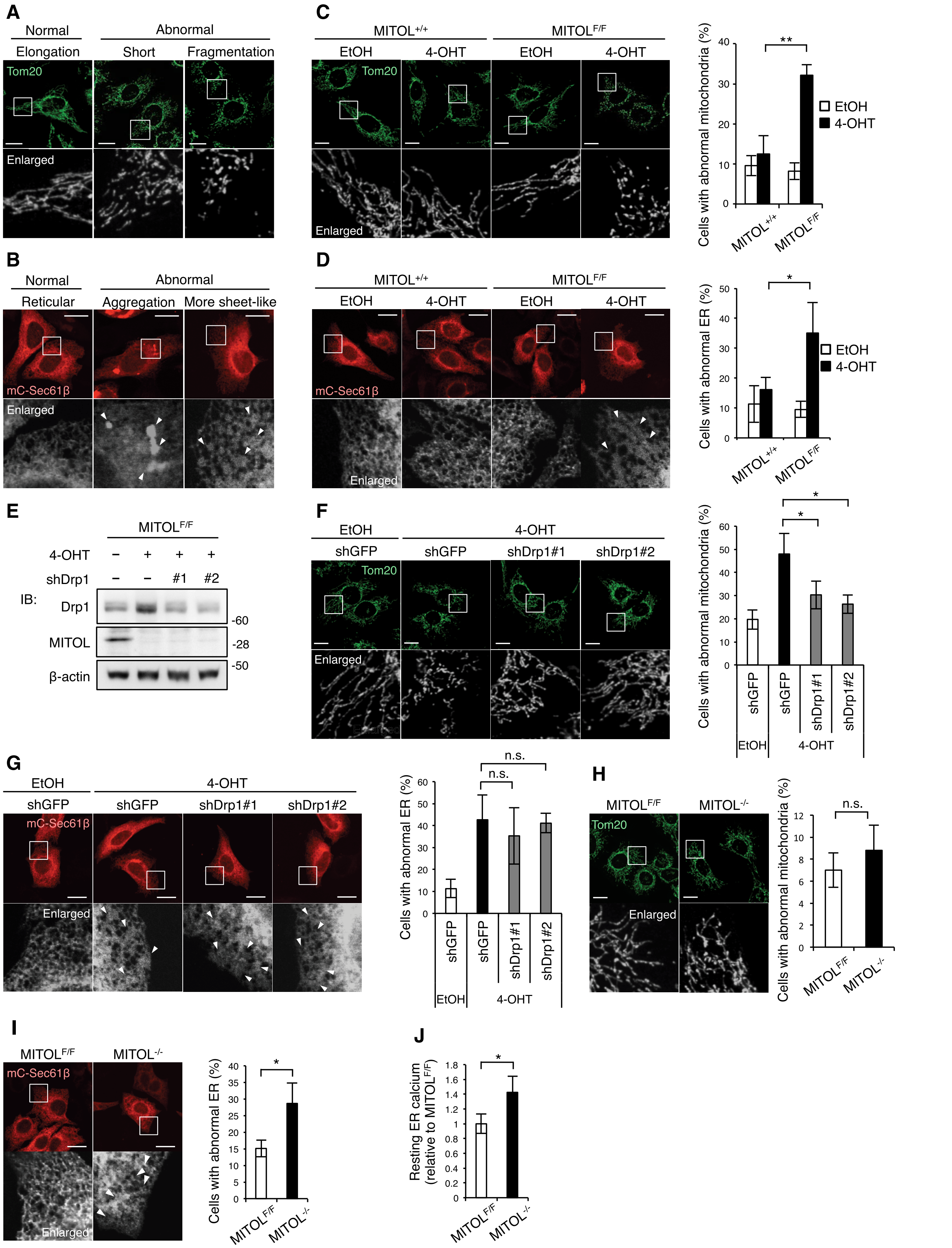

Figure EV1.

 © 2019 The Authors

Figure EV2. MITOL is dispensable for the PERK and ATF6 pathway and prevents IRE1α hyper-activation independent of its function in Drp1 regulation, related to Figs 2 and 3.

A MITOL depletion did not affect PERK autophosphorylation under ER stress. MEFs were treated with 0.7 mg/ml tunicamycin (Tu) for indicated periods. Error bars represent SD (n = 3). All experiments using Tu below were performed at concentration of 0.7 μg/ml. n.s.: not significant (Student's t-test).

B MITOL depletion did not affect the transcriptional activity of ATF6. MEFs were treated with Tu for indicated periods, followed by immunoblotting with indicated antibodies. cATF6: cleaved ATF6. ATF6 FL: ATF6 full length. Error bars represent SD (n = 3). n.s.: not significant (Student's t-test).

C, D MITOL depletion did not affect ER stress-induced *chop*, *atf4*, and *dr5* expressions. MEFs were treated with Tu for 4 h (C) and indicated periods (D). The levels of mRNA expression were monitored by qRT–PCR. Error bars represent SD (n = 3). n.s.: not significant (Student's t-test).

E JNK silencing rescued ER stress-induced apoptosis of MITOL-KO MEFs. MEFs were treated with scramble RNA or siJNK (#1, #2) 24 h prior to Tu treatment for 24 h, followed by immunoblotting with indicated antibodies. Error bars represent SD (n = 3). $^{**}P < 0.01$, $^{***}P < 0.001$ (Student's t-test).

F, G shDrp1 reduced morphological abnormalities of mitochondria in MITOL-KO MEFs. MEFs were transfected with shGFP or shDrp1#2 vector 24 h prior to Tu treatment for 4 h. These cells were analyzed by immunoblot analysis with indicated antibodies (F) or immunofluorescent analysis with anti-Tom20 antibody (G). The lower panels show threefold magnification image of the boxed regions. Scale bar represents 10 μm. Error bars represent SD (n = 6). $^{*}P < 0.05$, $^{***}P < 0.001$. n.s.: not significant (Student's t-test).

H–J shDrp1 did not affect IRE1α RNase activity under ER stress. Levels of mRNA expression were monitored by qRT–PCR. Error bars represent SD (n = 3). n.s.: not significant (Student's t-test).

K, L MITOL was not involved in PERK autophosphorylation and ATF6 cleavage after wash out of Tu. MEFs were treated with Tu for 4 h and washed with PBS and re-fed with fresh media for indicated periods, followed by immunoblotting with indicated antibodies. cATF6: cleaved ATF6.

© 2019 The Authors

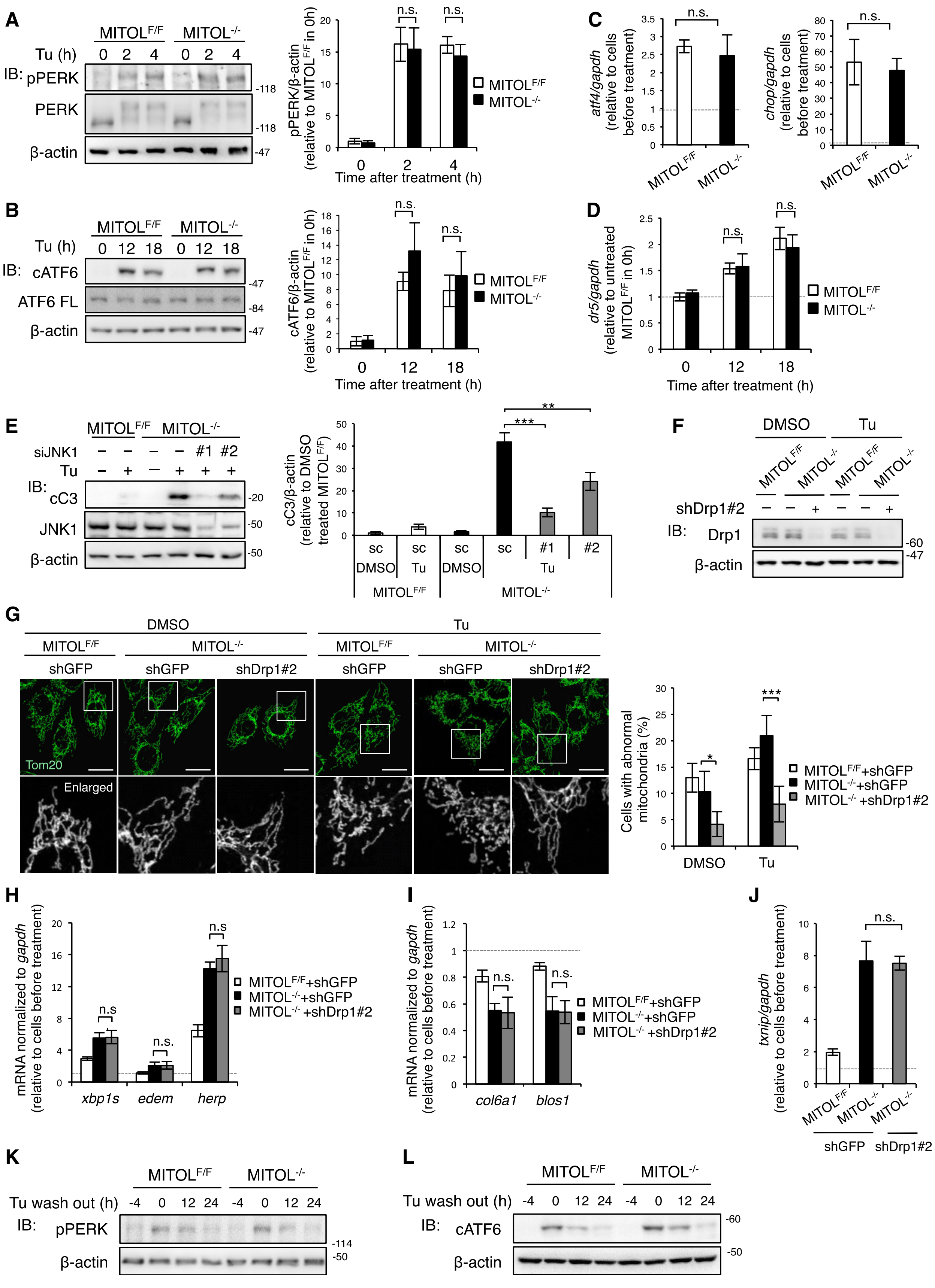

Figure EV2.

© 2019 The Authors

Figure EV3. MITOL ubiquitylates an inactivate form of IRE1α, related to Fig 4.

A *In vitro* ubiquitylation of IRE1α by MITOL. *In vitro* ubiquitylation assay was performed as described in methods. Immunoprecipitated IRE1α-FLAG from HEK293 cells was incubated with or without indicated materials, followed by immunoblotting with indicated antibodies.

B MITOL did not affect the protein turnover of IRE1α. MEFs were incubated with 10 μg/ml cycloheximide (CHX) for indicated periods, followed by immunoblotting with indicated antibodies. Error bars represent SD (n = 3). n.s.: not significant (Student's t-test).

C, D ER stress attenuated IRE1α ubiquitylation by MITOL and enhanced the interaction between IRE1α and BIM. MITOL-KO MEFs were transfected with indicated vectors 24 h prior to Tu treatment for indicated periods. Cell lysates were subjected to immunoprecipitated with indicated antibodies, followed by immunoblotting with indicated antibodies.

E The levels of cleaved caspase-3 of MEFs after Tu treatment. MEFs were transfected with empty vector or MITOL-coding vector 24 h prior to Tu treatment. Tu was treated for indicated periods, followed by immunoblotting with indicated antibodies.

F Autoubiquitylation activity of MITOL was not changed under ER stress. HEK293 cells were transfected with indicated vectors 24 h prior to Tu treatment for 12 h, followed by IP-IB analysis with indicated antibodies.

G MITOL preferentially ubiquitylated IRE1α monomer. HEK293 cells were transfected with indicated vectors, followed by IP-IB analysis with indicated antibodies. K121Y (KY) or D123P (DP) mutant of IRE1α inhibits its self-association.

H–J Inhibition of IRE1α RNase enhanced MITOL-dependent IRE1α ubiquitylation. HEK293 cells were transfected with indicated vectors 24 h prior to incubation with either 10 μM 4μ8C, 1 μM KIRA6, or 2 μM APY-29 for 3 h, followed by IP-IB analysis with indicated antibodies.

© 2019 The Authors

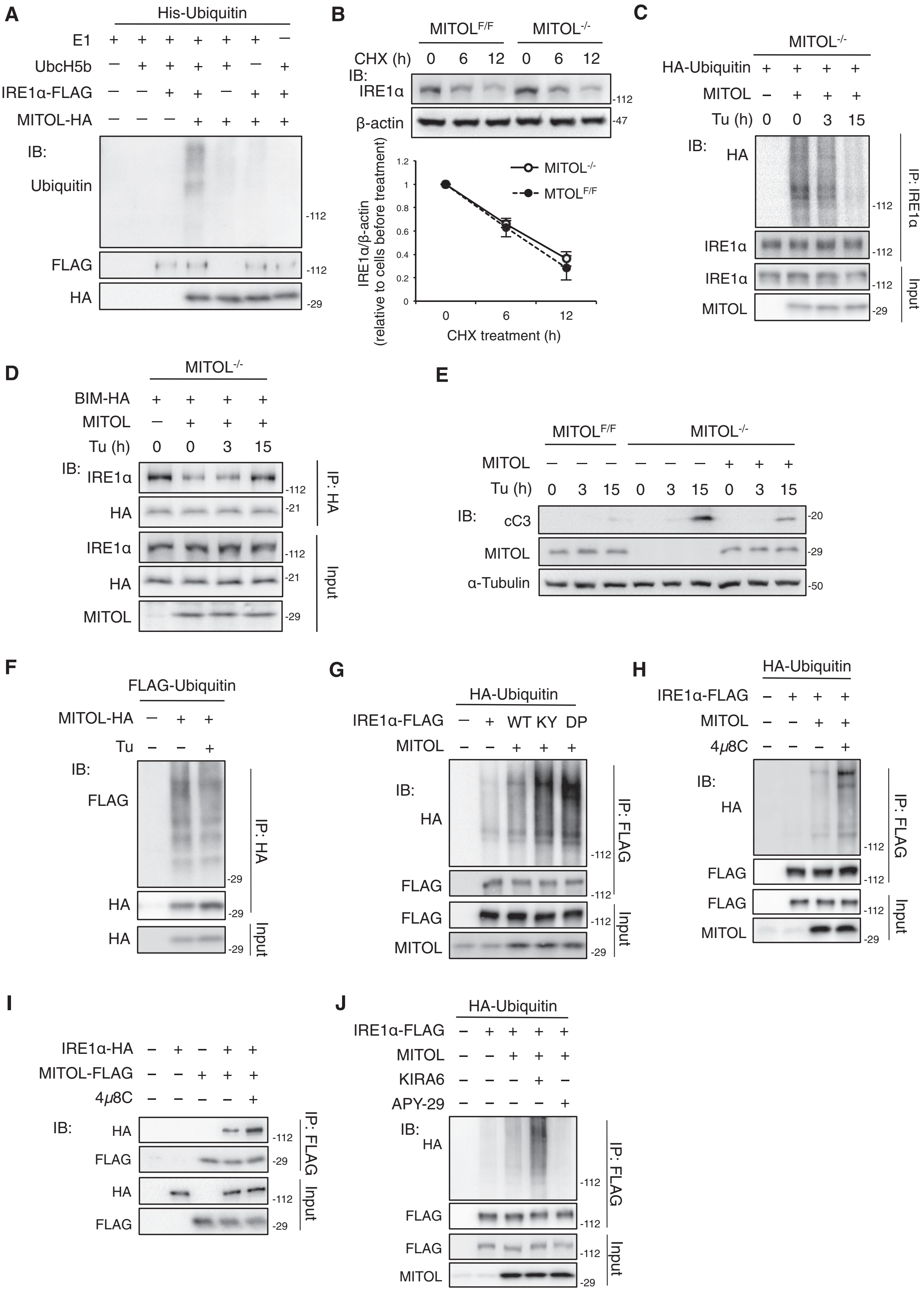

Figure EV3.

 © 2019 The Authors

Figure EV4. Defect of IRE1α ubiquitylation by MITOL leads to abnormal ER morphology, related to Fig 5.

A K481R mutation of IRE1α did not impair its RNase activity. IRE1α-KO MEFs were transfected with indicated vectors 24 h prior to Tu treatment for 4 h. Expression levels of *xbp1s* mRNA were determined by qRT–PCR. Error bars represent SD (n = 3). *P < 0.05, n.s.: not significant (Student's t-test).

B Overexpression of IRE1α K481R did not affect PERK and ATF6 pathway. MEFs were transfected with indicated vectors 24 h prior to immunoblotting with indicated antibodies. MEFs treated with Tu for 8 h were prepared as a positive control for UPR activation. cATF6: cleaved ATF6.

C, D The K481R mutation of IRE1α also induced cell death under ER stress. MEFs were transfected with indicated vectors 24 h prior to analysis. Viable cells were evaluated as similar to Fig 1A (C). Dead cells were quantified by cell toxicity assay using cytotoxicity LDH assay Kit-WST. Vec: empty vector. Error bars represent SD (n = 4). *P < 0.05, **P < 0.01, ***P < 0.001 (Student's t-test).

E Overexpression of IRE1α K481R-induced morphological change in the ER. MEFs were transfected with indicated vectors and mC-Sec61β 24 h prior to analysis. The lower panels show fivefold magnification image of the boxed regions. Arrowheads indicate abnormal parts of the ER network. Scale bar represents 10 μm. Error bars represent SD (n = 3). *P < 0.05 (Student's t-test).

F Overexpression of IRE1α K481R increased resting Ca^2 in the ER. MEFs were transfected with G-CEPIA1er 24 h before analysis. Resting Ca^{2+} in the ER was calculated from 10 cells in each independent experiment. Error bars represent SD (n = 3). *P < 0.05 (Student's t-test).

© 2019 The Authors

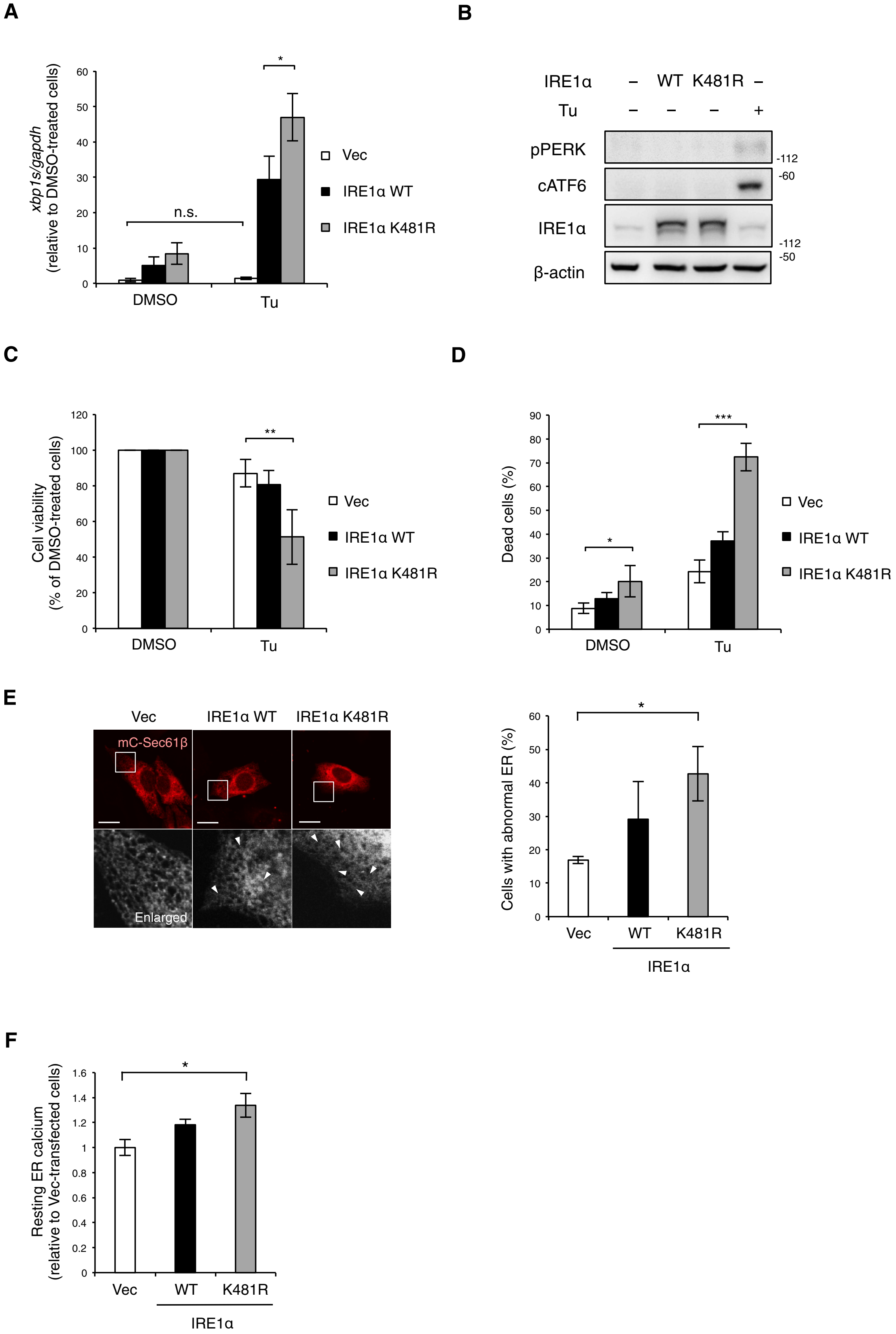

Figure EV4.

© 2019 The Authors

Figure EV5. IRE1α was localized at MAM and MITOL depletion does not affect PERK pathway in the spinal cord, related to Figs 6 and 7.

A Co-localization of MITOL with IRE1α. COS-7 cells were transfected with indicated vectors prior to immunostained with anti-HA or anti-FLAG antibody. The intensity profiles following a dotted line were performed using ImageJ. The lower panels show sevenfold magnification images of the boxed regions. Arrowheads indicate co-localization points of two signals. Scale bar represents 10 μm.

B, C MITOL and the K481R mutation of IRE1α did not alter the localization at MAM. Crude mitochondrial fraction containing MAM (Mito plus MAM) and the cytosolic fraction containing ER (Cyto plus ER) were isolated from MEFs (B) or MEFs transfected with indicated vectors (C), followed by immunoblotting with indicated antibodies.

D–F Generation of nerve-specific MITOL-KO mice. LoxP sequence inserted into the introns 2 and 3 of the *march5/mitol* locus. Nestin-Cre transgenic mice were crossed with these mice to generate mice lacking MITOL in the central and peripheral nervous system (D). mRNA and protein expression levels of MITOL in the spinal cord were confirmed by qRT–PCR (E) and immunoblotting (F).

G, H MITOL depletion did not change the activity of PERK in the spinal cord. Three-month-old mice were treated with 1 mg/kg Tu for indicated periods. Spinal cord was subjected to immunoblotting (G) or qRT–PCR (H). Error bars represent SD (n = 3). n.s.: not significant (Student's t-test).

© 2019 The Authors

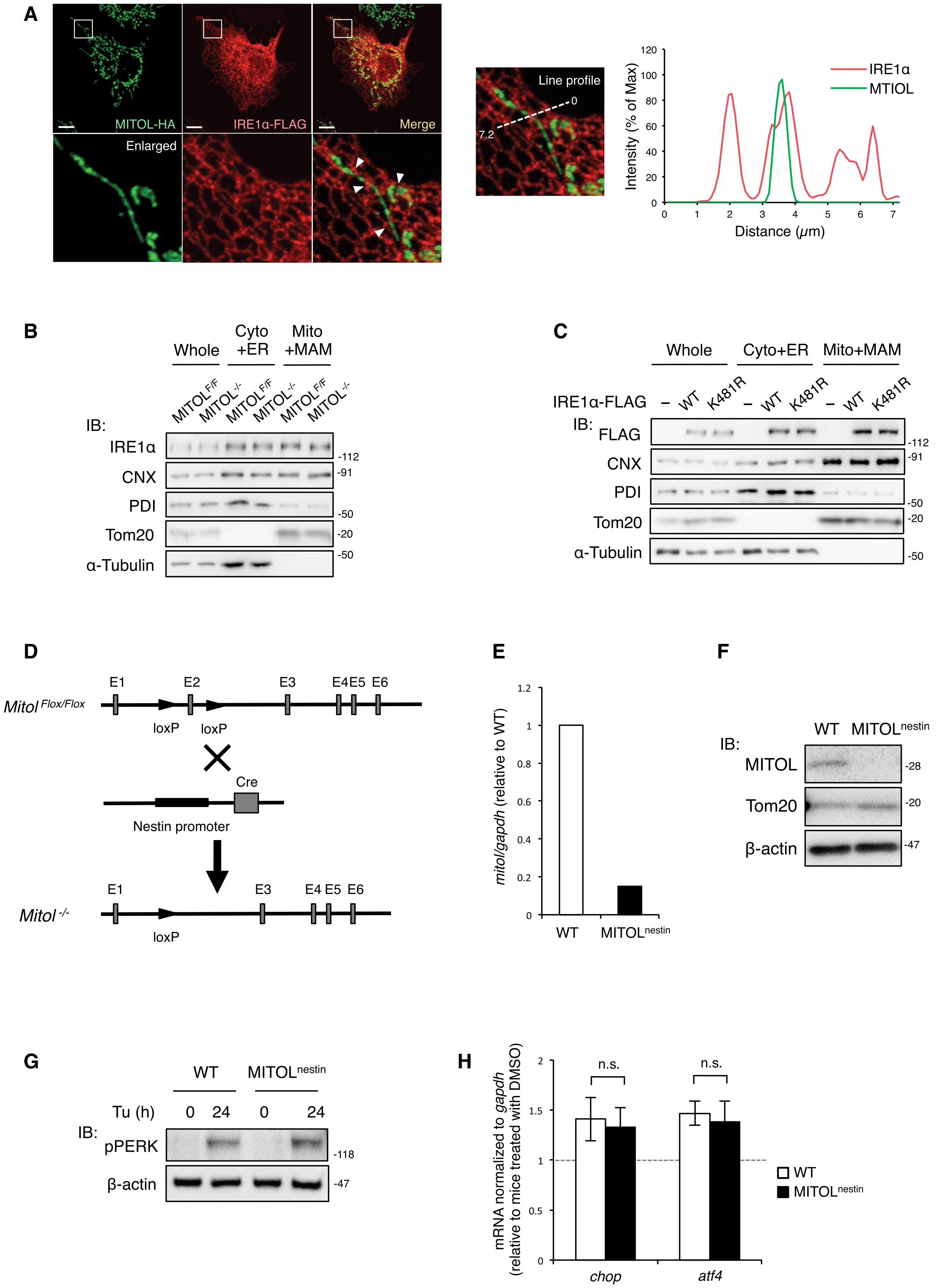

Figure EV5.

 © 2019 The Authors
